# Evaluation of the Efficacy of Stem Cell Therapy in Animal Models of Intervertebral Disc Degeneration Based on Imaging Indicators: A Systematic Review and Meta-Analysis

**DOI:** 10.1155/2022/2482653

**Published:** 2022-08-31

**Authors:** Wenhao Li, He Zhao, Zhencheng Xiong, Chuanhong Li, Jianbin Guan, Tao Liu, Yongdong Yang, Xing Yu

**Affiliations:** ^1^Dongzhimen Hospital Affiliated to Beijing University of Chinese Medicine, Beijing 100700, China; ^2^China-Japan Friendship Hospital, Beijing 100029, China

## Abstract

**Objective:**

The purpose of this study is to make a systematic review of the therapeutic effect of stem cells in animal models of disc degeneration from an imaging point of view.

**Methods:**

Data were extracted by searching electronic databases for RCTs that met the inclusion criteria. Data analysis was performed using RevMan 5.3 and STATA 15.1 software. This meta-analysis was registered with INPLASY, registration number INPLASY202240148.

**Results:**

A total of 34 studies were included, covering four species of animals, rabbits, sheep, rats, and mice, with a total of 1163 intervertebral discs. In terms of DHI, the efficacy of stem cell group in rabbits (*P* < 0.001), mice (*P* < 0.001), sheep (*P* < 0.001), and rats (*P* = 0.001) was better than that in control group. In terms of disc height, the efficacy of stem cell group in rats (*P* < 0.001) was better than that in control group, while in sheep (*P* = 0.355), there was no statistical difference between two groups. In terms of MRI index, the efficacy of stem cell group in rats (*P* < 0.001), mice (*P* < 0.001), and rabbits (*P* = 0.016) was better than that in control group. In terms of MRI signal score, the efficacy of stem cell group in rabbits (*P* < 0.001) was better than that of control group. In terms of T2 signal intensity, stem cell group was more effective than control group in rabbits (*P* < 0.001), mice (*P* < 0.001), and rats (*P* = 0.003).

**Conclusion:**

Stem cell therapy can improve intervertebral disc-related imaging parameters in animal models of disc degeneration, indicating that stem cell therapy has a repairing effect on intervertebral discs. However, given the heterogeneity and limitations of this study, this conclusion still needs to be tested by a large number of studies.

## 1. Introduction

Degenerative disc disease is a clinical syndrome caused by permanent structural changes in the intervertebral disc, and it is one of the most common clinical diagnoses [1]. It has been reported that more than 50% of asymptomatic patients between the ages of 30 and 39 have disc degeneration and loss of disc height [2]. According to statistics, there are 1 million lumbar disc surgeries worldwide each year, and only 10% and 15% of lumbar disc degeneration require surgical treatment [3]. Disc degeneration imposes a heavy economic and medical burden on the global aging population [4].

Disc degeneration is a complex multifactorial process determined by genetic, nutritional, and mechanical factors [5]. It is characterized by loss of intradiscal cells and extracellular matrix, upregulation of matrix metalloproteinases (MMPs), and inflammatory mediators, resulting in irreversible damage to the disc structure [6]. Current conservative and surgical treatments focus on relieving symptoms rather than preventing degeneration or restoring disc structure and function [7]. Surgery may even exacerbate the degeneration of adjacent healthy discs [1]. Therefore, there is an urgent need for a regenerative therapy to repair degenerated discs.

In the past 20 years, people have tried to find new breakthroughs in the treatment of disc degeneration from stem cells. Encouragingly, these efforts are paying off. There has been a growing body of research demonstrating the therapeutic potential of stem cells in animal models, and clinical studies are taking the first steps [1, 8–10]. Since no one has yet summarized and evaluated this, we designed this study to systematically evaluate the therapeutic effect of stem cell therapy in animal models from an imaging point of view.

## 2. Methods

This study was conducted according to the Preferred Reporting Items for Systematic Reviews and Meta-Analyses (PRISMA) statement [11] and the Cochrane Handbook [12]. Because this study was a systematic review of published researches, it did not require ethical approval. This meta-analysis was registered with INPLASY. The registration number is INPLASY202240148, and the DOI is 10.37766/inplasy2022.4.0148 [13].

### 2.1. Inclusion and Exclusion Criteria

Studies that met the following requirements were included: (1) randomized controlled trials (RCTs) using animal models of disc degeneration as the research object, and the animal species were not limited; (2) stem cell therapy was used as an intervention measure, and other drugs were used as control measure. Both could be combined with other drugs or materials; (3) there were no restrictions on the source of stem cells, the dose of drugs, and the course of treatment.

Studies were excluded according to the following exclusion criteria: (1) the animal species used were different from other literatures and could not be compared with any of the other included studies; (2) the imaging evaluation indexes or imaging data representations used were different from other literatures and could not be compared with any other included studies.

### 2.2. Search Strategies

After determining the inclusion and exclusion criteria for this study, two researchers independently searched multiple databases, including PubMed, Cochrane Library, ScienceDirect, CNKI, and Wanfang Database. The retrieved articles were published before March 1, 2022. The following search terms were used: disc degeneration, animal model, stem cell, mesenchymal stem cell, bone marrow-derived mesenchymal stem cell, adipose-derived stem cell, MSC, BMSC, and ADSC with the Boolean operators AND or OR. The retrieved literatures were screened by two researchers step by step according to title, abstract, and full text. After identifying included articles, we traced their references to identify potential articles.

### 2.3. Data Extraction

After screening was complete, data were extracted from eligible literatures by two independent researchers and then cross-checked by a third researcher. For the differences in the included literatures, all researchers reached consensuses through discussion. The data extracted in this study included the name of the first author, year of publication, country, animal species, modeling method, stem cell type, stem cell source, injection dose, interventions in the control group, and imaging evaluation indicators.

### 2.4. Quality Assessment

Since the target literatures for this study were RCTs in animals, we used the Cochrane Risk Bias Tool [14] for quality assessment. This work was done using Review Manager software (RevMan 5.3).

### 2.5. Data Analysis

We performed statistical analysis of data extracted from each study using STATA software (version 15.1). Continuous variables were reported as mean difference (MD) and 95% confidence interval (CI), while dichotomous variables were reported as odds ratio (OR) and 95% CI. Statistical heterogeneity was judged according to the *I*^2^ statistic. The greater the *I*^2^, the greater the heterogeneity. If there was heterogeneity in this study (*I*^2^≥50%), a random-effects model was used; otherwise, a fixed-effects model (*I*^2^<50%) was used. In this study, differences were considered statistically significant when *P* < 0.05.

## 3. Results

### 3.1. Search Result

**Figure 1:**
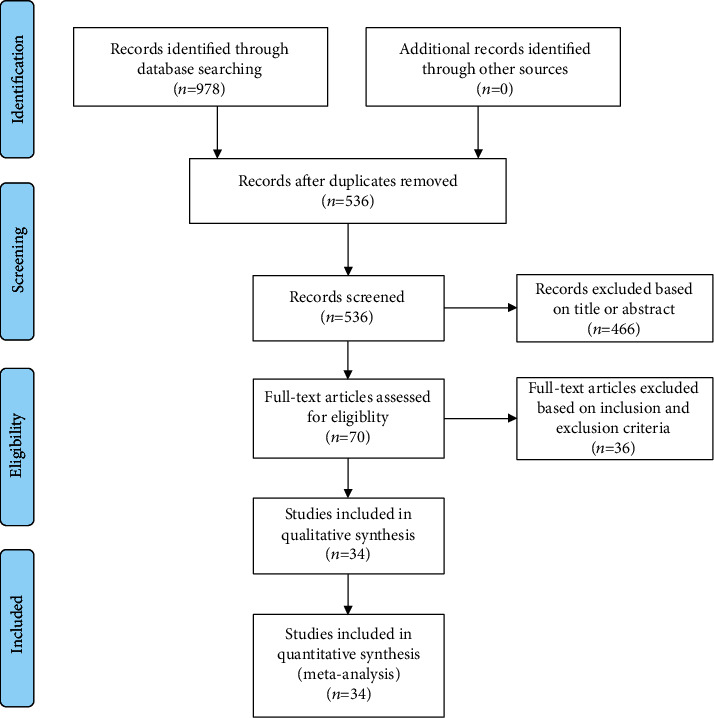
Flow chart of literature screening.

According to the above search strategies, 978 relevant articles were preliminarily identified, including PubMed (*n* = 136), Cochrane Library (*n* = 2), ScienceDirect (*n* = 354), CNKI (*n* = 400), and Wanfang Database (*n* = 86). After removing duplicate studies, 34 studies were finally included according to the inclusion and exclusion criteria. The flow chart of literature screening is shown in Figure 1, and the basic characteristics of the included studies are shown in Table 1.

### 3.2. Quality Assessment

**Figure 2:**
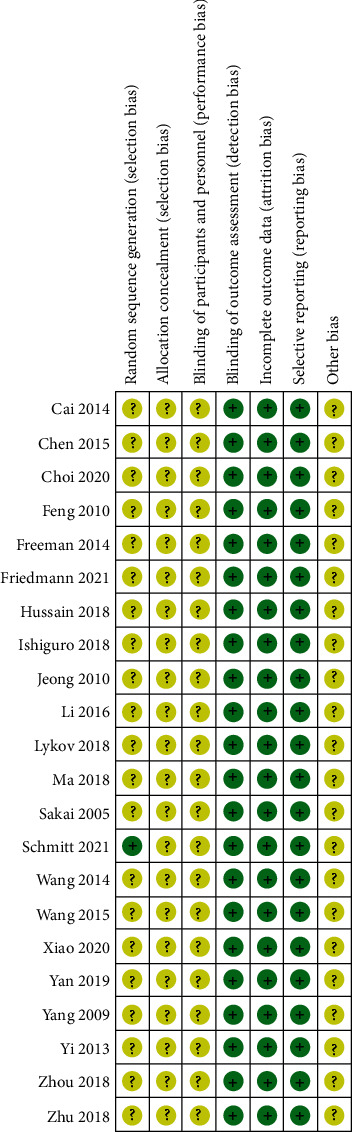
Risk of bias summary. +: low risk of bias; ?: bias unclear.

In the included studies, except Schmitt et al. [8] used a random number generator to generate random sequences, the rest of the studies did not explain how random sequences were generated. None of the studies mentioned blinding and allocation concealment. No selective reporting and incomplete data were found in all studies. The existence of other biases could not be determined as shown in Figure 2.

### 3.3. Results of the Meta-Analysis

#### 3.3.1. DHI

**Figure 3:**
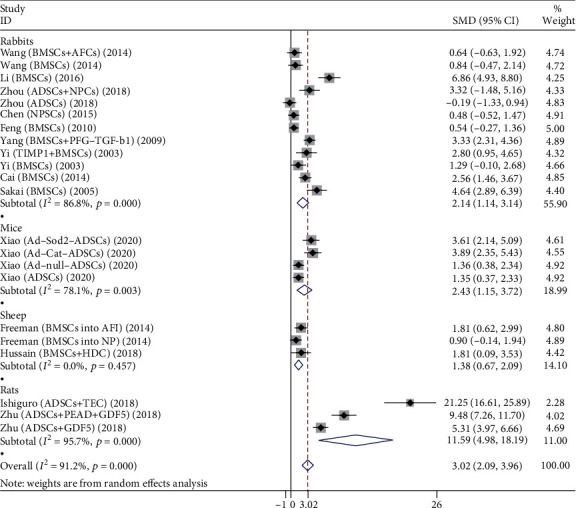
Forest plot showing the effect of stem cell group and control group on DHI in animal models.

A total of 22 studies compare the DHI of the stem cell group and the control group, as shown in Figure 3, including rabbits, rats, mice, and sheep.

Twelve studies evaluated the effects of both groups in rabbits, including 214 rabbit discs. The main types of stem cells are BMSCs, ADSCs, and NPSCs. The heterogeneity test showed significant heterogeneity between studies (*P* < 0.001, *I*^2^ = 86.8%), so a random-effects model was used. The comprehensive results showed that the difference between the stem cell group and the control group was statistically significant (*P* < 0.001), and the stem cell group was better than the control group.

Four studies evaluated the effects of both groups in mice, involving 80 mouse discs. Stem cell types are mainly ADSCs. The heterogeneity test showed significant heterogeneity between studies (*P* = 0.003, *I*^2^ = 78.1%), so a random-effects model was used. The comprehensive results showed that the difference between two groups was statistically significant (*P* < 0.001), and the stem cell group was better than the control group.

Three studies evaluated the effects of both groups in sheep, involving 40 sheep discs. Stem cell types are mainly BMSCs. The heterogeneity test showed no significant heterogeneity between studies (*P* = 0.496, *I*^2^ = 0.0%), so a fixed-effects model was used. The comprehensive results showed that the difference between two groups was statistically significant (*P* < 0.001), and the stem cell group was better than the control group.

Three studies evaluated the effects of both groups in rats, including 123 rat discs. Stem cell types are mainly ADSCs. The heterogeneity test showed significant heterogeneity between studies (*P* < 0.001, *I*^2^ = 95.7%), so a random-effects model was used. The comprehensive results showed that the difference between two groups was statistically significant (*P* = 0.001), and the stem cell group was better than the control group.

#### 3.3.2. Disc Height

**Figure 4:**
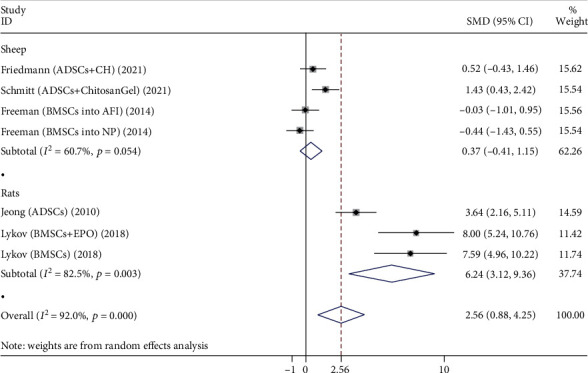
Forest plot showing the effect of stem cell group and control group on disc height in animal models.

A total of 7 studies compare the disc height of the stem cell group and the control group, as shown in Figure 4, including both sheep and rats.

Four studies evaluated the effects of both groups in sheep and included 70 sheep discs. The main types of stem cells are BMSCs and ADSCs. The heterogeneity test showed significant heterogeneity between studies (*P* = 0.054, *I*^2^ = 60.7%), so a random-effects model was used. The comprehensive results showed that the difference between two groups was not statistically significant (*P* = 0.355).

Three studies evaluated the effects of both groups in rats, involving 60 rat discs. The main types of stem cells are BMSCs and ADSCs. The heterogeneity test showed significant heterogeneity between studies (*P* = 0.003, *I*^2^ = 82.5%), so a random-effects model was used. The comprehensive results showed that the difference between two groups was statistically significant (*P* < 0.001), and the stem cell group was better than the control group.

#### 3.3.3. MRI Index

**Figure 5:**
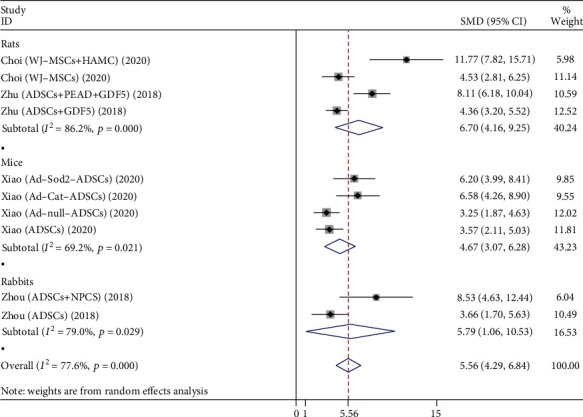
Forest plot showing the effect of stem cell group and control group on MRI index in animal models.

A total of 10 studies compare the MRI index of the stem cell group and the control group, as shown in Figure 5, including rats, mice, and rabbits.

Four studies evaluated the effects of both groups in rats, involving 120 rat discs. Stem cell types are mainly WJ-MSCs and ADSCs. The heterogeneity test showed significant heterogeneity between studies (*P* < 0.001, *I*^2^ = 86.2%), so a random-effects model was used. The comprehensive results showed that the difference between two groups was statistically significant (*P* < 0.001), and the stem cell group was better than the control group.

Four studies evaluated the effects of both groups in mice, involving 80 mouse discs. Stem cell types are mainly ADSCs. The heterogeneity test showed significant heterogeneity between studies (*P* = 0.021, *I*^2^ = 69.2%), so a random-effects model was used. The comprehensive results showed that the difference between two groups was statistically significant (*P* < 0.001), and the stem cell group was better than the control group.

Two studies evaluated the effects of both groups in rabbits and included 24 rabbit discs. Stem cell types are mainly ADSCs. The heterogeneity test showed significant heterogeneity between studies (*P* = 0.029, *I*^2^ = 79.0%), so a random-effects model was used. The comprehensive results showed that the difference between two groups was statistically significant (*P* = 0.016), and the stem cell group was better than the control group.

#### 3.3.4. MRI Signal Score

**Figure 6:**
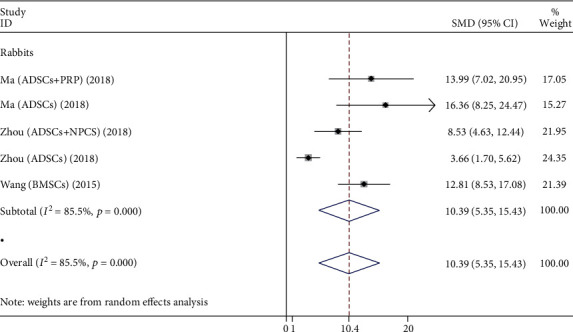
Forest plot showing the effect of stem cell group and control group on MRI signal score in animal models.

A total of 5 studies compared the MRI signal score of the stem cell group and the control group. As shown in Figure 6, there is only one animal, the rabbit, which contains 64 rabbit discs. Stem cell types are mainly BMSCs and ADSCs. The heterogeneity test showed significant heterogeneity between studies (*P* < 0.001, *I*^2^ = 85.5%), so a random-effects model was used. The comprehensive results showed that the difference between two groups was statistically significant (*P* < 0.001), and the stem cell group was better than the control group.

#### 3.3.5. T2 Signal Intensity

**Figure 7:**
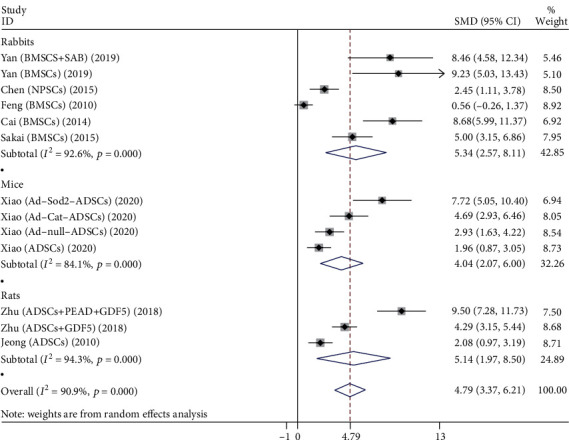
Forest plot showing the effect of stem cell group and control group on T2 signal intensity in animal models.

A total of 13 studies compare the T2 signal intensity of the stem cell group and the control group, as shown in Figure 7, including rabbits, mice, and rats.

Six studies evaluated the effects of both groups in rabbits, including 108 rabbit discs. Stem cell types are mainly BMSCs and NPSCs. The heterogeneity test showed significant heterogeneity between studies (*P* < 0.001, *I*^2^ = 92.6%), so a random-effects model was used. The comprehensive results showed that the difference between two groups was statistically significant (*P* < 0.001), and the stem cell group was better than the control group.

Four studies evaluated the effects of both groups in mice, involving 80 mouse discs. Stem cell types are mainly ADSCs. The heterogeneity test showed significant heterogeneity between studies (*P* < 0.001, *I*^2^ = 84.1%), so a random-effects model was used. The comprehensive results showed that the difference between two groups was statistically significant (*P* < 0.001), and the stem cell group was better than the control group.

Three studies evaluated the effects of both groups in rats, involving 100 rat discs. Stem cell types are mainly ADSCs. The heterogeneity test showed significant heterogeneity between studies (*P* < 0.001, *I*^2^ = 94.3%), so a random-effects model was used. The comprehensive results showed that the difference between two groups was statistically significant (*P* = 0.003), and the stem cell group was better than the control group.

#### 3.3.6. Publication Bias

**Figure 8:**
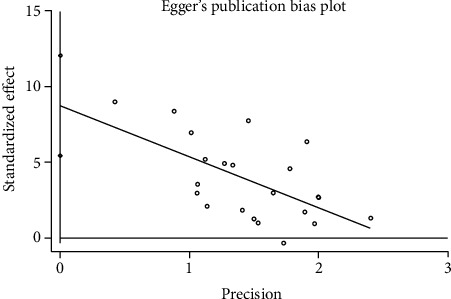
Publication bias of DHI.

**Figure 9:**
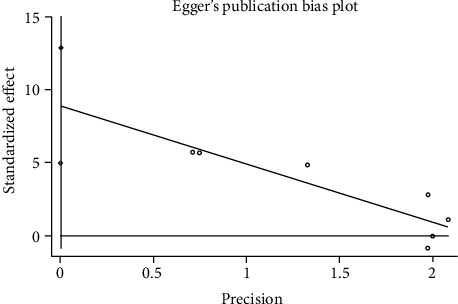
Publication bias of disc height.

**Figure 10:**
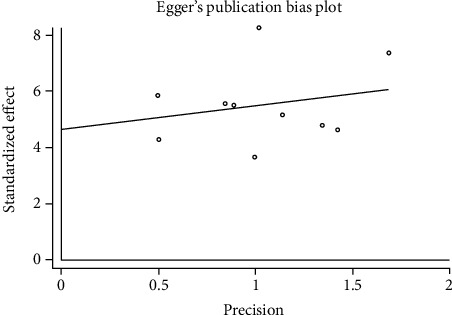
Publication bias of MRI index.

**Figure 11:**
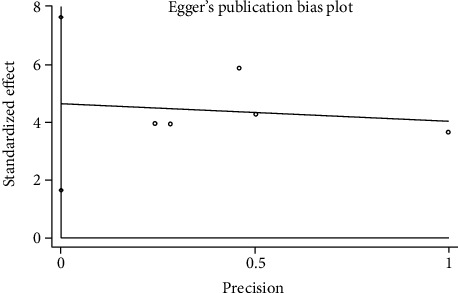
Publication bias of MRI signal score.

**Figure 12:**
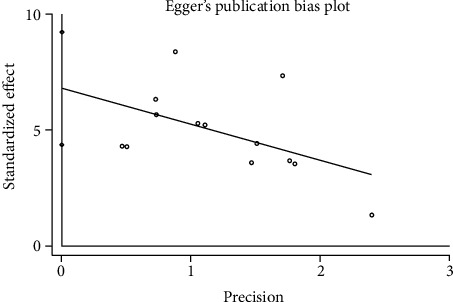
Publication bias of T2 signal intensity.

We used Egger's method to detect publication bias. The test results showed that DHI (*P* < 0.001), disc height (*P* = 0.002), MRI index (*P* = 0.01), MRI signal score (*P* = 0.016), and T2 signal intensity (*P* < 0.001) had publication bias (*P* < 0.05), as shown in Figures 8–12. We believe that publication bias may arise from the selective reporting and publication of positive results by authors and publishers.

#### 3.3.7. Sensitivity Analysis

**Figure 13:**
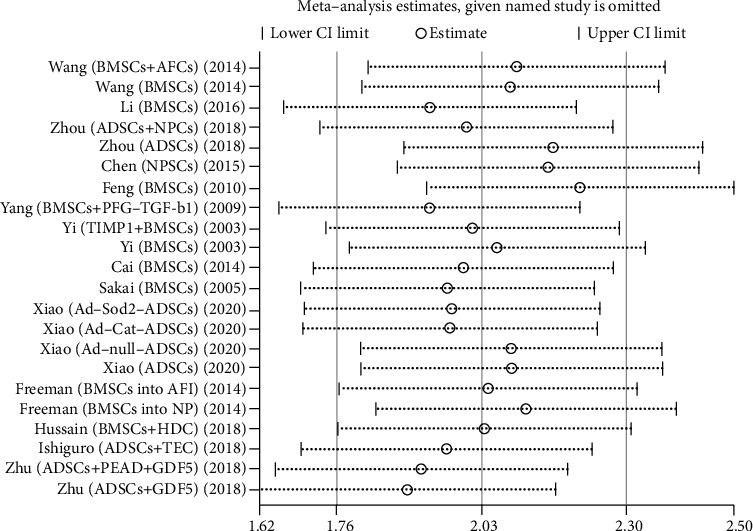
Sensitivity analysis of DHI.

**Figure 14:**
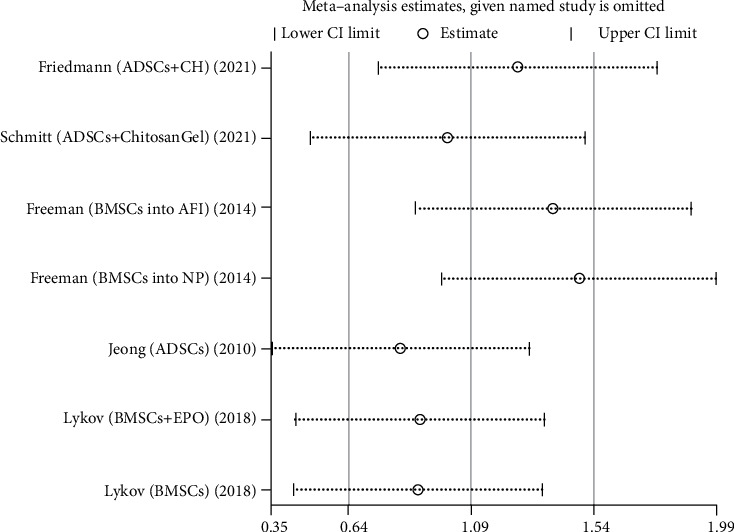
Sensitivity analysis of disc height.

**Figure 15:**
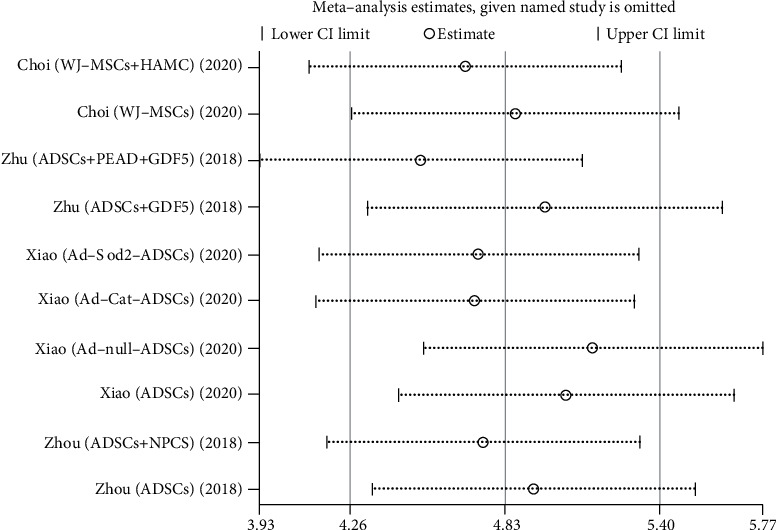
Sensitivity analysis of MRI index.

**Figure 16:**
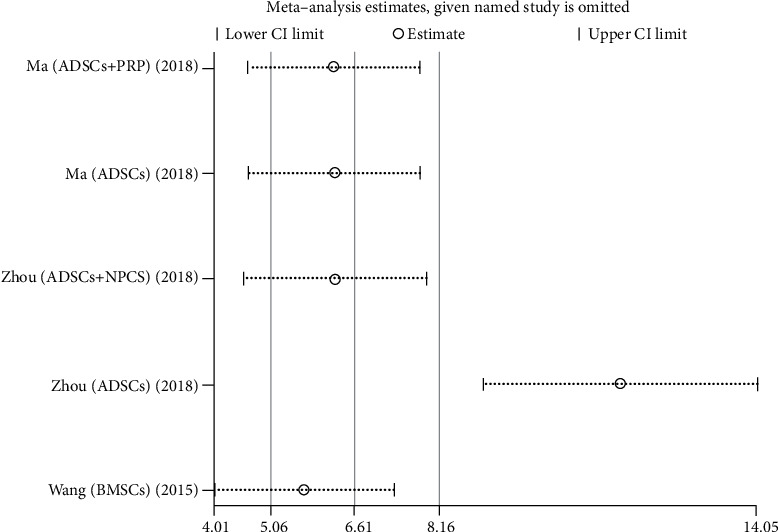
Sensitivity analysis of MRI signal score.

**Figure 17:**
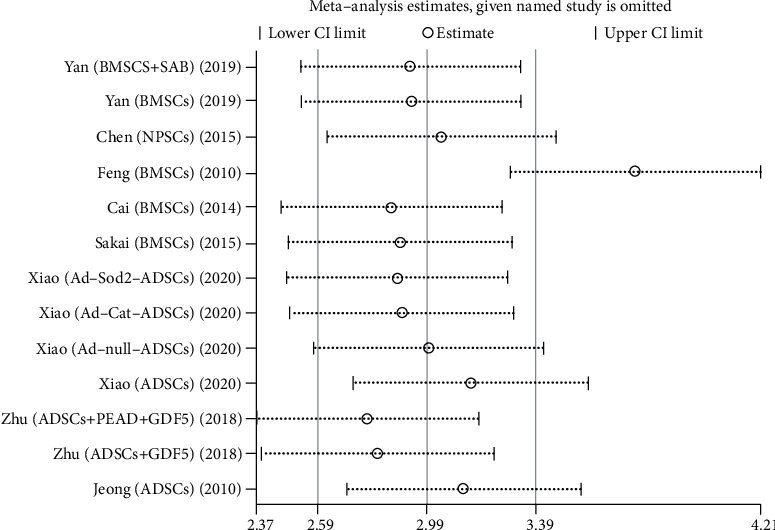
Sensitivity analysis of T2 signal intensity.

We conduct sensitivity analysis by excluding articles one by one, as shown in Figures 13–17. It can be seen from the figures that in the MRI signal score, after excluding the study of Zhou et al. [18], the combined results changed significantly. In T2 signal intensity, the combined results changed significantly after excluding the study by Feng et al. [25]. This indicates that these two studies may be one of the sources of heterogeneity. The remaining merger results are robust and reliable.

## 4. Discussion

In this meta-analysis, we included a total of 34 studies covering four species of animals: rabbits (410 discs), sheep (110 discs), rats (403 discs), and mice (240 discs). We used five imaging indicators, DHI, disc height, MRI index, MRI signal score, and T2 signal intensity, to evaluate the efficacy of stem cells in animal models. To our knowledge, this is the first study to systematically evaluate the efficacy of stem cells in animal models of disc degeneration over the past 20 years. In our study, the stem cell group covered both monotherapy and combination types, and subgroup analyses were performed to minimize heterogeneity and increase confidence in the results. Similar studies have not been done yet.

The results of our study showed that the effect of the stem cell group in rabbits, mice, sheep, and rats was better than that of the control group in terms of DHI. In terms of disc height, the efficacy of the stem cell group in rats was better than that in the control group, while in sheep, there was no statistical difference in efficacy between the two groups. In terms of MRI index, the effect of the stem cell group in rats, mice, and rabbits was better than that in the control group. In terms of MRI signal score, the stem cell group was better than the control group in rabbits. In terms of T2 signal intensity, the stem cell group was more effective than the control group in rabbits, mice, and rats.

Determining a suitable cell source is the premise and challenge for the successful establishment of disc regeneration therapy [33]. Despite initial success, treatments using purely autologous disc cell transplantation have not been satisfactory [34, 35]. In particular, disc degeneration can precede disc herniation [36, 37]. Therefore, there is a need for an alternative source of autologous cells. The repairing effect of stem cells in the intervertebral disc has been confirmed by a growing number of studies.

In the studies we included, ADSCs and BMSCs were the most commonly used stem cells, both of which are able to differentiate into chondrocytes [38, 39]. BMSCs not only differentiate themselves into nucleus pulposus cells, but also nourish the remaining nucleus pulposus cells by producing cytokines such as transforming growth factor-*β*1 (TGF-*β*1) [40, 41]. The effects of ADSCs are mainly reflected in reducing apoptosis, inhibiting pro-inflammatory factors, inhibiting catabolic factors, and promoting cell proliferation [42–45]. Since BMSCs only account for a small part of bone marrow cells, tissue damage is inevitable during the extraction process [1]. In contrast, ADSCs can be easily collected from adipose tissue with a lower complication rate, and the yield is higher than that of BMSCs, so they are more acceptable [1, 8].

Although the above studies have confirmed that stem cell therapy can repair the intervertebral disc, it still has a limitation; that is, the content of type II collagen and proteoglycan in the repaired intervertebral disc is still lower than that of the normal intervertebral disc [22]. Therefore, from the characteristics of the studies we included (Table 1), it can be seen that in the past 20 years, stem cell therapy has undergone a process from being used alone to being used in combination with other drugs or novel biomaterial scaffolds. The application of new biomaterial scaffolds has gradually attracted the attention of scholars. It has been suggested that injecting stem cells into degenerated discs alone is not enough, as acidic environment and inflammation can inhibit their proliferation, while hypoxia and nutrient deprivation may lead to apoptosis [46]. In contrast, scaffolds are designed to provide a suitable three-dimensional microenvironment for injected cells without cytotoxicity, allowing their distribution and proliferation and promoting cell survival and differentiation [9]. At the same time, the adhesion provided by the scaffold can limit the leakage of cells through the annulus fibrosus fissure, avoiding osteophyte formation and potential nerve root compression [8]. This is the theoretical basis for the use of bioscaffolds. However, there is no optimal bioscaffold material yet [9]. From the studies we included, hydrogels reinforced with various biomaterials (such as collagen, hyaluronic acid, and chitosan) have been widely used due to their high biocompatibility, high viscosity, and biodegradability. Although the hydrogel can provide some mechanical stability, its elastic modulus is still lower than that of healthy nucleus pulposus tissue, so it cannot provide sufficient mechanical support for the intervertebral disc [18]. Therefore, in addition to simulating the local biological environment, enhancing mechanical properties is another important goal of bioscaffold research [18]. Of course, the safety and long-term efficacy of these novel bioscaffolds also remains to be verified over time.

From the perspective of the construction method of animal models, acupuncture caused annulus fibrosus damage, which was widely used in the included studies. This modeling method has the advantages of simple operation, low cost, and strong repeatability, and can cause the degeneration of the intervertebral disc in a relatively short period of time. However, the disadvantage is that there is a risk of infection, which may cause an immune inflammatory response [47]. Also, as previously discussed, annulus fibrosus fissures caused by acupuncture may lead to cell leakage. From the point of view of animal selection, although it is believed that large animals, especially sheep, because of the absence of notochordal cells and the good comparability with human intervertebral discs in terms of biomechanical properties, are the best simulations of human intervertebral discs except primates [47]. However, from the studies we included, rabbits are still the most used animals, followed by rats, sheep, and mice. We believe that this may be related to the economic cost of animals. Large animals are more expensive to raise and require higher experimental facilities. During the literatures search, we found that there were also a small number of studies using canine, porcine, and rhesus monkey models, but due to the small number and no common outcome indicators, meta-analysis could not be performed, so these studies could only be excluded. We believe that, when economic conditions permit, large animals are still the ideal animal choice for disc degeneration. Although rabbits are not as effective in simulating human intervertebral discs as large animals, they have larger intervertebral discs compared to rats and mice, are easy to operate, and have strong tolerance. They are still a suitable animal choice for limited economic conditions. It should be pointed out that, given the complexity of human disc degeneration, there is no animal model that can fully simulate the entire pathophysiological process of human intervertebral disc, and a perfect animal model of disc degeneration still does not exist [47].

Imaging evaluation is the most commonly used clinical evaluation method for disc degeneration. In terms of imaging indicators, MRI index, MRI signal score, T2 signal intensity, DHI, and disc height were the imaging indicators commonly used in the included studies (Table 1). Therefore, we performed this meta-analysis using these five indicators. In addition to this, there were also studies using indicators such as Pfirrmann grade, endplate degeneration score, NP mid-sectional volume, and NP T2 relaxation time to assess the degree of disc degeneration. Unfortunately, the amount of literature using these metrics was too small to perform a meta-analysis, so we had to discard these metrics. Since the loss of intervertebral height is the most common imaging manifestation of disc degeneration, DHI and disc height were used in most studies, and most of them were measured on X-ray films. Disc height is a direct measurement method, which is greatly affected by factors such as population, age, gender, height, weight, and body position, and cannot obtain personalized measurement results. Therefore, the concept of DHI was proposed, which focuses on reflecting the changes in the disc height relative to the adjacent vertebral bodies, and is a more personalized indicator [48]. The included studies also reflect this, with DHI being used by more studies relative to disc height. X-ray inspection has the advantages of fast, convenient, and low cost. However, it cannot directly image the intervertebral disc and is suitable for quick, rough assessment. Although MRI is expensive and time-consuming, it can directly observe the intervertebral disc, and the signal intensity can directly reflect the degree of disc degeneration. MRI index, MRI signal score, and T2 signal intensity are MRI-related indicators. In the studies we included, these three indicators were directly calculated with the help of third-party imaging software, such as Image J, GE ADW work station, Analyze Direct, Paravision, and other software. Except for the MRI index (NP area multiplied by the mean signal intensity [49]), no studies have described the detailed calculation of MRI signal score and T2 signal intensity. We speculate that this may be related to the different ways of data representation adopted by different software. To ensure the accuracy of the results, we did not combine these two indicators for analysis. In conclusion, from the studies we included, disc height and MRI signal intensity are still widely used imaging indicators for evaluating disc degeneration.

Our study has the following limitations. First, the heterogeneity of the studies is large. We believe that this may be related to the large differences in the injected dose of cells and the observation time between each study. Moreover, the imaging equipment used differs between studies, and the specific values of the images are calculated by third-party software, and the types of these softwares are also different, which may bias the results. Another point is the restriction of animal movement in the rearing environment, which was mentioned in only a few of the studies we included. We believe that differences in the amount of animal exercise caused by crowded and loose housing environments may also affect the degenerative process of the intervertebral disc, which is also a source of heterogeneity. Finally, we have yet to find studies examining the mechanical properties of the repaired disc, which may be the next step for refinement.

## 5. Conclusion and Prospect

In this study, through a large-scale meta-analysis of 34 studies, under multiple animal species and multiple evaluation indicators, the therapeutic effect of stem cells in the animal models of disc degeneration was observed from the perspective of imaging, which further demonstrated the role of stem cells in promoting intervertebral disc repair. However, it should be pointed out that it is not enough to demonstrate this repair effect from an imaging perspective alone. Researches on histology, biomechanics, and other aspects are also essential, which is also the direction of our next efforts.

At present, most of the studies related to the repair of disc degeneration with stem cells are still in the stage of cell and animal experiments, and there is still a distance from clinical use. Encouragingly, there are already studies taking the first steps towards clinical application [50–52]. In addition, the current bioscaffold materials still have much room for improvement in terms of biocompatibility and mechanical properties. Finally, how to deliver stem cells more safely and accurately into the body and the timing of intervention are still issues that need to be resolved in the future.

## Figures and Tables

**Table 1:** Characteristics of the studies included.

Author	Year	Country	Study type	Animal	Modeling method	Types of stem cells	Source of stem cells	Injection dose	Control	Observation time	Imaging-based evaluation indicators
Friedmann et al. [1]	2021	Germany	RCT	Merino sheep, female	Nucleotomy	ADSCs+collagen hydrogel	Sheep	5.5 × 10^4^	Not provided	12 months	Disc height
Schmitt et al. [8]	2021	Germany	RCT	Merino sheep, female	Nucleotomy	ADSCs+chitosan carboxymethyl cellulose hydrogel scaffold	Sheep	5 × 10^6^	Not provided	12 months	Disc height
Choi et al. (1) [9]	2020	Korea	RCT	Sprague-Dawley rats, female	Needle puncture	WJ-MSCs+HAMC	Human	2 × 10^4^	PBS	6 weeks	MRI index
Choi et al. (2) [9]						WJ-MSCs					
Xiao et al. (1) [10]	2020	China	RCT	Mice	Needle puncture	Sod2-ADSCs	Human	1 × 10^5^	Not provided	8 weeks	DHI MRI index T2 signal intensity
Xiao et al. (2) [10]						Cat-ADSCs					
Xiao et al. (3) [10]						Null-ADSCs					
Xiao et al. (4) [10]						ADSCs					
Yan et al. (1) [15]	2019	China	RCT	New Zealand white rabbits	Nucleus pulposus aspiration	BMSCs+SAB	Rabbits	1.75 × 10^5^	PBS	8 weeks	T2 signal intensity
Yan et al. (2) [15]						BMSCs					
Ma et al. (1) [16]	2018	China	RCT	New Zealand white rabbits	Needle puncture	ADSCs+PRP	Human	1 × 10^7^	PBS	4 weeks	MRI signal score
Ma et al. (2) [16]						ADSCs					
Zhou et al. (1) [17]	2018	China	RCT	New Zealand white rabbits	Needle puncture	ADSCs+NPCS	Human	6 × 10^4^	Not provided	16 weeks	DHI MRI index
Zhou et al. (2) [17]						ADSCs					
Ishiguro et al. [18]	2018	Japan	RCT	Sprague-Dawley rats, male	Nucleotomy	ADSCs+TEC	Rats	6 × 10^5^	NP reimplantation	6 months	DHI Endplate degeneration score
Hussain et al. [19]	2018	United States	RCT	Finn sheep	Nucleotomy	BMSCs+HDC	Sheep	2.5 × 10^5^	HDC	6 weeks	DHI Pfirrmann grade NP mid-sectional volume NP T2 relaxation time
Zhu et al. (1) [20]	2018	China	RCT	Sprague-Dawley rats, male	Needle puncture	ADSCs+PEAD+GDF5	Human	Not provided	PEAD+ GDF5	24 weeks	DHI MRI index T2 signal intensity
Zhu et al. (2) [20]						ADSCs+GDF5					
Lykov et al. (1) [21]	2018	Russia	RCT	Wistar rats, male	Needle puncture	BMSCs+EPO	Rats	1 × 10^5^	Not provided	3 weeks	Disc height
Lykov et al. (2) [21]						BMSCs					
Li et al. [22]	2016	China	RCT	New Zealand white rabbits	Nucleotomy	BMSCs+DSP	Rabbits	1 × 10^4^	DSP	12 weeks	DHI
Wang et al. [23]	2015	China	RCT	New Zealand white rabbits, male	Needle puncture	BMSCs+PRP	Sheep	2 × 10^5^	PRP	8 weeks	MRI signal score
Chen et al. [24]	2015	China	RCT	New Zealand white rabbits, male	Nucleus pulposus aspiration	NPSCs	Human	1 × 10^6^	NPCs	2 months	DHI T2 signal intensity
Wang et al. (1) [25]	2014	China	RCT	New Zealand white rabbits	Nucleus pulposus aspiration	BMSCs+AFCs	Rabbits	Not provided	Saline	2 weeks	Disc height ratio Pfirrmann grade
Wang et al. (2) [25]						BMSCs					
Freeman et al. (1) [26]	2014	Australia	RCT	Merino wether sheep	Nucleotomy	BMSCs into AF	Sheep	1 × 10^6^	PBS	12 months	DHI Disc height Pfirrmann grade
Freeman et al. (2) [26]						BMSCs into NP					
Cai et al. [27]	2014	China	RCT	New Zealand white rabbits	Needle puncture	BMSCs	Rabbits	2 × 10^4^	PBS	10 weeks	T2 signal intensity T2 relaxation time DHI
Yi et al. (1) [28]	2013	China	RCT	New Zealand white rabbits	Needle puncture	TIMP1-BMSCs	Rabbits	3 × 10^7^	PBS	12 weeks	DHI
Yi et al. (2) [28]						BMSCs					
Feng et al. [29]	2010	China	RCT	New Zealand white rabbits	Nucleus pulposus aspiration	BMSCs	Rabbits	1 × 10^6^	NPCs	16 weeks	DHI T2 signal intensity
Jeong et al. [30]	2010	Korea	RCT	Sprague-Dawley rats, female	Needle puncture	ADSCs	Human	5 × 10^4^	Saline	6 weeks	Disc height T2 signal intensity
Yang et al. [31]	2009	China	RCT	New Zealand white rabbits	Nucleus pulposus aspiration	BMSCs+PFG-TGF-b1	Rabbits	2 × 10^6^	PFG-TGF-b1	12 weeks	DHI
Sakai et al. [32]	2005	Japan	RCT	New Zealand white rabbits	Nucleus pulposus aspiration	BMSCs	Rabbits	4 × 10^4^	Atelocollagen	24 weeks	T2 signal intensity DHI

WJ-MSCs: Wharton's jelly-derived mesenchymal stromal cells; HAMC: hyaluronan–methylcellulose; PBS: phosphate-buffered saline; BMSCs: bone marrow-derived mesenchymal stem cells; AFCs: annulus fibrosus cells; ADSCs: adipose-derived mesenchymal stem cells; PRP: platelet-rich plasma; DSP: dexamethasone sodium phosphate; NP: nucleus pulposus; AF: annulus fibrosus; NPCS: decellularized nucleus pulposus-based cell delivery system; TEC: scaffold-free tissue-engineered construct; HDC: riboflavin cross-linked high-density collagen gel; DHI: disc height index; SAB: salvianolic acid B; Ad-null: ADSCs transduced with an adenovirus vector containing no transgene expression cassette; Ad-Sod2: ADSCs transduced with recombinant adenovirus Sod2; Ad-Cat: ADSCs transduced with recombinant adenovirus Cat; PEAD: a growth factor delivery vehicle composed of heparin and the synthetic polycation poly(ethylene argininylaspartate diglyceride); GDF5: growth and differentiation factor-5; NPSCs: Nucleus pulposus-derived stem cells; PFG-TGF-b1: pure fibrinous gelatin-transforming growth factor-b1; EPO: erythropoietin; TIMP: tissue inhibitor of metalloproteinases.

## Data Availability

All data generated or analyzed during this study are included in this article.

## Abbreviations

MMPs: Matrix metalloproteinases
PRISMA: Preferred reporting items for systematic reviews and meta-analyses
RCT: Randomized controlled trial
MD: Mean differences
OR: Odds ratios
CI: Confidence intervals
CNKI: China National Knowledge Infrastructure
WJ-MSCs: Wharton's jelly-derived mesenchymal stromal cells
HAMC: Hyaluronan-methylcellulose
PBS: Phosphate-buffered saline
BMSCs: Bone marrow-derived mesenchymal stem cells
AFCs: Annulus fibrosus cells
ADSC: Adipose-derived mesenchymal stem cells
PRP: Platelet-rich plasma
DSP: Dexamethasone sodium phosphate
NP: Nucleus pulposus
AF: Annulus fibrosus
NPCS: Decellularized nucleus pulposus-based cell delivery system
TEC: Scaffold-free tissue-engineered construct
HDC: Riboflavin cross-linked high-density collagen gel
DHI: Disc height index
SAB: Salvianolic acid B
Ad-null: ADSCs transduced with an adenovirus vector containing no transgene expression cassette
Ad-Sod2: ADSCs transduced with recombinant adenovirus Sod2
Ad-Cat: ADSCs transduced with recombinant adenovirus cat
PEAD: A growth factor delivery vehicle composed of heparin and the synthetic polycation poly(ethylene argininylaspartate diglyceride)
GDF5: Growth and differentiation factor-5
NPSCs: Nucleus pulposus-derived stem cells
PFG-TGF-b1: Pure fibrinous gelatin-transforming growth factor-b1
EPO: Erythropoietin
TIMP: Tissue inhibitor of metalloproteinases.
